# Impact of Human Epidermal Growth Factor Receptor 2 (HER2) Low Status in Response to Neoadjuvant Chemotherapy in Early Breast Cancer

**DOI:** 10.7759/cureus.22330

**Published:** 2022-02-17

**Authors:** Fátima R Alves, Lucia Gil, Leonor Vasconcelos de Matos, Ana Baleiras, Carolina Vasques, Maria Teresa Neves, André Ferreira, Mário Fontes-Sousa, Helena Miranda, Ana Martins

**Affiliations:** 1 Medical Oncology, Centro Hospitalar de Lisboa Ocidental, Lisbon, PRT; 2 Medical Oncology, Centro Hospitalar Universitário de Lisboa Central, Lisbon, PRT; 3 Medical Oncology, Fundação Champalimaud, Lisbon, PRT; 4 Medical Oncology, Hospital CUF Tejo, Lisbon, PRT

**Keywords:** trastuzumab-deruxtecan, trastuzumab-duocarmazine, antibody-drug conjugate, her2 negative, her2 low breast cancer, her2 breast cancer

## Abstract

Introduction

In clinical practice, there is a binary distinction between human epidermal growth factor receptor 2 (HER2)-positive and HER2-negative (HER2−) breast cancer (BC). However, within HER2− disease, there is significant heterogeneity. Particularly, HER2− tumors that express some level of HER2 by immunohistochemistry (IHC) score 1+ or 2+/in situ hybridization (ISH) non-amplified are currently defined as HER2-low. This subgroup has shown distinct biological features compared to HER2-zero (HER2-0) BC and additionally novel antibody-drug conjugate therapies have demonstrated a potential and promising activity in HER2-low BC population. This study aims to evaluate the impact of HER2-low status in response to neoadjuvant chemotherapy (NACT) in HER2− BC being HER2-low and HER2-0 status.

Materials and methods

In a single institution, we retrospectively reviewed clinical and pathological data of HER2 early-stage BC patients treated with NACT following definitive surgery from January 2015 to December 2020. Tumors with HER2 IHC 0 were classified as HER2-0 and IHC score 1+ and 2+/ISH non-amplified as HER2-low. The primary objective was to evaluate the rate of pathological complete response (pCR) using the definition of ypT0/Tis ypN0 according to HER2-low and HER2-0 subgroups. Secondary objectives were to evaluate biological features between the two subgroups, disease-free survival (DFS), and overall survival (OS). Pearson chi-square, Fisher’s exact, and Mann-Whitney tests were performed. The Kaplan-Meier method was used to plot DFS and OS curves. A p-value of <0.05 was considered statistically significant.

Results

A total of 72 patients with HER2 BC were included with a median age at diagnosis of 52.5 years and a median follow-up time of 35.5 months. Of patients, 56.9% had HER2-low disease and 43.1% had HER2-0 disease. Significant differences between the two subgroups were detected regarding hormonal receptor status and proliferation grade (Ki67). In the HER2-low subgroup, 70% of tumors were luminal-like and 64.5% of HER2-0 patients had triple-negative BC (p* *= 0.03). There were statistically significant differences regarding estrogen (p = 0.00) and progesterone (p = 0.02) receptors. The median Ki67 rate was higher in the HER2-0 subset (mean rank = 43.9) compared to HER2-low (mean rank = 30.9) and this difference was statistically significant (p* *= 0.00). HER2-low patients presented more stage III tumors (65.9%) and HER2-0 patients were mainly stage II (61.3%), and this was statistically relevant (p* *= 0.03). The prevalence of other clinical and pathological features was comparable between both groups. HER2-low subgroup achieved lower pCR rates (14.6% vs.* *29.0%) but this difference was not statistically significant (p* *= 0.15). Similarly, there was no difference between the two subgroups regarding DFS (p* *= 0.97) and OS (p* *= 0.35), although the data were immature.

Conclusion

As in prior studies, this study did not support a significant impact of HER2-low status on response to NACT in HER2− patients with early-stage BC. HER2-low patients had a lower pCR, which may suggest a worse response to classic chemotherapy regimen and may have clinical implications that should be further exploited. The prevalence of hormonal receptors in HER2-low tumors was consistent with previous data in the literature. Although retrospective, the data suggest that HER2-low tumors should be regarded as a distinct biological subtype and more research is warranted.

## Introduction

Breast cancer (BC) is a heterogeneous disease and the most frequently diagnosed carcinoma in women worldwide [1]. It comprises particular biologic entities that differ in prognosis and oncogenic factors. Therapeutic choices are usually based on traditional histopathological features, with the recognition in the clinical practice of four main subtypes of BC with predictive and prognostic value: luminal A-like, luminal B-like (human epidermal growth factor receptor 2 (HER2)-negative), HER2-positive (HER2+), and triple-negative BC (TNBC) [1].

More particularly, HER2+ BC represents 15-20% of all breast tumors and is defined by the Erb-B2 receptor tyrosine kinase 2 (ERBB2)/HER2 gene amplification that leads to overexpression of the transmembrane tyrosine kinase receptor protein [1,2]. These aspects are associated with more aggressive behavior, which leads to a decreased overall survival (OS) and disease-free survival (DFS), if untreated. However, the development and institution of multiple agents targeting HER2 in the medical practice have provided substantial clinical benefits, reducing the risk of recurrence in the early stage and/or improving survival in the advanced setting [1,2]. Regardless of therapeutic advances, about 4-23% of patients with the localized disease still face recurrence after (neo)adjuvant anti-HER2 treatments. In the metastatic setting, median survival rates have systematically improved in recent years, reaching a median of ~57 months, although it may be even longer for patients receiving novel therapies [2,3].

Based on the 2018 American Society of Clinical Oncology (ASCO)/College of American Pathologists (CAP) guideline update, HER2+ BC is clinically characterized by a complete and intense circumferential membrane staining for the HER2 protein in >10% of tumor cells (3+ score) at immunohistochemistry (IHC) and/or in situ hybridization (ISH)-based techniques with a HER2/chromosome enumeration probe 17 (CEP17) ratio ≥ 2.0 and an average HER2 gene copy number ≥ 4.0 signals/cell [4]. And so, tumors with IHC 0, 1+, or 2+/ISH negative are defined as HER2 negative (HER2−). Therefore, in clinical practice, there is a binary distinction between HER2+ and HER2− BC. However, within HER2− disease, significant heterogeneity exists and the current HER2 definition does not suitably consider HER2 disease′s clinical and biological variations [5,6]. In fact, a meaningful proportion (45-55%) of tumors that are classified as HER2− show a low or moderate expression of HER2 without ERBB2 amplification, recently named “HER2-low BC” [7,8]. Indeed, within HER2− BC, 65% of hormone receptor-positive (HR+)/HER2− tumors and 37% of TNBC are HER2-low [2]. The HER2-low BC subtype represents a new recently proposed nomenclature for those tumors with an IHC assay score of 1+ or 2+ but with a negative ISH assay [6,8].

The HER2-low subgroup is currently not targetable with standard anti-HER2 agents as demonstrated in the National Surgical Adjuvant Breast and Bowel Project (NSABP) B47 trial, which showed no clinical benefit by adding trastuzumab, an anti-HER2 targeted therapy, to adjuvant chemotherapy (CT) in the high-risk population [7,9]. However, more recent clinical data from non-randomized trials have shown a potential advantage of new anti-HER2 therapies in HER2-low pretreated patients and have yielded promising results [10]. These findings show that antibody-drug conjugates (ADC), in particular, trastuzumab deruxtecan [11,12] and trastuzumab duocarmazine [13], may have clinical activity in tumors that have low to moderate levels of HER2 expression beside classical HER2+ BC, revealing objective response rates ranging between 32% and 37% in the HER2-low population [7,11-13].

This has resulted in the presumption that HER2-low BC might constitute a different disease category, from other luminal and TNBC, with particular clinical characteristics and therefore potential for novel targeted therapies [14]. Consequently, this can be especially demanding for clinical pathologists, who are mostly focused on identifying HER2+ tumors (IHC score 3+ or IHC 2+/ISH positive) [7]. On the other hand, the definition of HER2-low inherently depends on the testing procedure and currently can only be performed with the conventional IHC/ISH techniques, since specific parameters that would define a tumor as HER2-low through other analyses have not been properly established [1].

Currently, little is known about HER2-low BC and prognostic data are scarce. In the early setting, despite no evidence have shown a potential benefit for anti-HER2 targeted agents in the HER2-low population so far, this subset of patients might also benefit from targeting HER2. Limited data have been reported on the impact of HER2-low expression on response to CT. In a pooled analysis of four prospective, neoadjuvant clinical trials, Denkert et al. have identified HER2-low as a distinct subgroup from HER2-0, with specific biology and differences in response to therapy and prognosis [7]. Pathological complete response (pCR) has been demonstrated by a large pooled analysis to have a strong association with improved long-term benefit as measured by DFS and OS, in triple-negative and HER2+ BC [15]. Exploring the impact of HER2 status (HER2-low vs. HER2-0) in achieving pCR would add up to tailoring neoadjuvant treatment strategies to improve outcomes for this subgroup of patients.

Therefore, more data on the HER2-low population are urgently needed to clarify the clinical and molecular characteristics of this subgroup and to allow the integration of new drugs into treatment guidelines. The purpose of this study is to evaluate the differential impact of HER2-low status in rates of pCR after neoadjuvant chemotherapy (NACT) in early BC.

## Materials and methods

Study design and population

This is a single institutional retrospective and observational study with the intent of evaluating the predictive value of HER2-low status in achieving pCR defined as no invasive residual carcinoma in breast and lymph nodes (ypT0/Tis ypN0), and subsequent prognostic impact, after NACT in early BC. It included all early-stage HER2− BC patients who underwent NACT followed by surgery at our hospital (Centro Hospitalar de Lisboa Ocidental, Portugal) between January 2015 and December 2020.

Our eligibility criteria were as follows: age >18 years old, pre-treatment biopsy compatible with invasive breast carcinoma, HER2 IHC score 0, 1+, or 2+/ISH negative, treatment with NACT followed by surgery with curative intent, and hormonal receptors could be either positive or negative. Patients with HER2+ BC (IHC 3+ or IHC 2+ ISH amplified) or IHC 2+ ISH equivocal, clinical IV, history of bilateral or previous invasive breast carcinoma, or other primary neoplasia history were excluded. Insufficient data about tumor pathological features or treatment received and exclusive neoadjuvant treatment with radiotherapy (RT) or hormonal therapy (HT) were also excluded.

Hormone receptor (HR) status and HER2 status were characterized before any treatment on biopsy specimens. HER2 status was determined according to ASCO/CAP guidelines available at the time of diagnosis: HER2-low tumors were defined by IHC as 1+ or IHC 2+/ISH non-amplified. HER2-0 was defined as IHC 0. HR status was characterized based on IHC data currently available: luminal-like if estrogen receptor and/or progesterone were ≥1% or TNBC if estrogen <1% and progesterone receptor <1%; and histologic grade according to the Nottingham histologic scoring system.

For our patient’s selection, we assessed the pathological database and identified 986 BC patients who underwent surgery, of which 179 were after NACT. Of these, 75 were HER2+ diseases. A total of 72 eligible patients were included in the final analysis. The patient flowchart is presented in the Appendix for additional information.

Medical electronic records and pathology reports were reviewed and information on clinical, pathological, and treatment data were gathered, according to pre-determined categories, comprising gender, date of birth and diagnosis, Eastern Cooperative Oncology Group (ECOG) performance status (previous to treatment), menopausal status, histologic subtype, differentiation grade, proliferation index (Ki67), tumor node metastasis (TNM) staging, HER2 and HR status, treatments’ data received as NACT (including regimen), neoadjuvant RT or HT, date and type of surgery, pCR, margin resection, and residual cancer burden (RCB).

Data regarding clinical outcomes on recurrence and survival were also obtained: DFS was defined as the time from last treatment (surgery) until any relapse assessed by Response Evaluation Criteria in Solid Tumors (RECIST) 1.1 or censor date and OS was defined as the time from diagnosis to death, irrespective of cause or censor date; distant or local recurrence was also obtained. This study was approved by the institutional Ethics Committee in accordance with the Declaration of Helsinki.

Study objectives

The primary objective of this study was to determine the rate of pCR according to HER2-low and HER2-0 status. Thus, we hypothesized that the pCR rate may differ according to HER2 status, in patients with HR+ or hormone receptor-negative (HR−) and HER2− disease undergoing NACT.

Our secondary objectives were to evaluate differential tumor biology features and staging, according to HER2 status (HER2-low vs. HER2-0) and to evaluate the impact of low HER2 expression on DFS assessed at six and 12 months by RECIST 1.1 and OS assessed at six and 12 months.

Statistical analysis

The statistical analysis was generated using Statistical Package for the Social Sciences (SPSS®) software, version 24.0 (IBM Corp., Armonk, NY). Data were reported descriptively (relative and absolute frequencies) for each of the pre-determined parameters as presented above. Categorical variables were represented as proportions and continuous variables as mean ± SD or median (interquartile range) as appropriate. Pearson χ² test was used to correlate categorical parameters with more than two categories. Chi-square test or Fisher's exact test were used to explore differences in the frequency of categorical variables between the HER2-low and HER-0 groups. Mann-Whitney test was applied to correlate continuous variables and T-test was applied to compare the normally distributed continuous variables. Survival curves, OS, and DFS, were plotted by the Kaplan-Meier method and compared using the log-rank test. A p-value of < 0.05 was considered statistically significant.

## Results

Baseline clinical and pathological characteristics as well as the treatments offered are summarized in Table 1. Of the 72 HER2− patients included from January 2015 to December 2020, one patient was male and 71 were females, the median age at diagnosis was 52.5 years (32-86 years), and median follow-up time was 35.5 months (95% CI, 10-75 months). The number of HER2-low patients was 41 (56.9%) and 31 (43.1%) were HER2-0. Among HER2-low patients, 18 (43.9%) score IHC 1+ and 23 (56.1%) score IHC 2+/ISH non-amplified.

**Table 1:** Clinical and pathological characteristics stratified by HER2-low status and HER2-0 status. * Categorical variables were compared by Pearson χ² test (parameters with more than two categories), Fisher’s exact test (for binary parameters), and the Mann-Whitney test (for continuous parameters). IQR – interquartile range; SLNB – sentinel lymph node biopsy; CNS – central nervous system; CT – chemotherapy; RT – radiotherapy; HT – hormonal therapy; ECOG – Eastern Cooperative Oncology Group; HR – hormone receptor; TNBC – triple-negative breast cancer.

Characteristics	HER2-low (n = 41)	HER2-0 (n = 31)	P-value*
Median age (range)	53 (32-86)	50 (34-80)	0.34
Age IQR (median)	29 (43-72)	17 (44-61)	
Pretreatment ECOG performance status (%)			
0	38 (92.7)	29 (93.5)	1.00
1	3 (7.3)	2 (6.5)	
Menopausal status (%)			
Pre/perimenopausal	17 (41.5)	14 (45.2))	1.00
Postmenopausal	23 (56.1)	17 (54.8)	
Male	1 (2.4)	0 (0)	
Histology (%)			
Ductal/non-special type	31(75.6)	26 (83.9)	0.75
Lobular	6 (14.6)	3 (9.7)	
Other subtypes	4 (9.8)	2 (6.5)	
Differentiation grade (%)			
I	9 (22)	1 (3.2)	0.07
II	19 (46.3)	17 (54.8)	
III	13 (31.7)	13 (41.9)	
Hormonal receptors (%)			
HR-negative (TNBC)	12 (29.3)	20 (64.5)	0.03
HR-positive (luminal)	29 (70.7)	11 (35.5)	
Estrogen receptor (mean rank)	41 (43.9)	31 (26.8)	0.00
Progesterone receptor (mean rank)	41 (40.7)	31 (30.9)	0.02
Ki67 (mean rank)	41 (30.9)	31 (43.9)	0.00
Ki67 subgroups (%)			
≤20	10 (24.4)	4 (12.9)	0.22
>20	31 (75.6)	27 (87.1)	
cT (%)			
T1	1 (2,4)	0 (0.0)	0.39
T2	16 (39.0)	16 (51.6)	
T3	15 (36.6)	12 (38.7)	
T4	9 (22.0)	3 (9.7)	
cN (%)			
N0	13 (31.7)	16 (51.6)	0.16
N1	19 (46.3)	7 (22.6)	
N2	7 (17.1)	5 (16.1)	
N3	2 (4.9)	3 (9.7)	
Clinical stage (%)			
I	0 (0.0)	0 (0.0)	0.03
II	14 (34.1)	19 (61.3)	
III	27 (65.9)	12 (38.7)	
Neoadjuvant CT regimen (%)			
Anthracycline +/- taxane	35 (85.4)	30 (96.8)	0.30
No anthracycline or taxane	4 (9.8)	0 (0.0)	
Other regimens	2 (4.8)	1 (3.2)	
Dose-dense regimen (%)			
No	28 (68.3)	14 (45.2)	0.58
Yes	13 (31.7)	17 (54.8)	
Neoadjuvant RT (%)			
No	40 (97.6)	30 (96.8)	1.00
Yes	1 (2.4)	1 (3.2)	
Neoadjuvant HT (%)			
No	41 (100)	29 (96.7)	0.42
Yes	0 (0.0)	1 (3.3)	
Surgery (%)			
Mastectomy + SLNB	6 (14.6)	7 (22.6)	0.20
Modified radical mastectomy	26 (63.4)	13 (41.9)	
Tumorectomy + SLNB	8 (19.5)	11 (35.5)	
Tumorectomy +axillary dissection	1 (2.4)	0 (0.0)	
yPT (%)			
T0	6 (14.6)	8 (25.8)	0.42
T1	16 (39)	11 (35.5)	
T2	10 (24.4)	6 (19.4)	
T3	7 (5.7)	3 (9.7)	
T4	1 (2.4)	0 (0.0)	
Tis	1 (2.4)	3 (9.7)	
ypN (%)			
N0	18 (43.9)	21 (67.7)	0.27
N1	11 (26.8)	5 (16.1)	
N2	8 (19.5)	3 (9.7)	
N3	4 (9.8)	2 (6.5)	
Pathological complete response (pCR) (%)			
No	35 (85.4)	22 (71.0)	0.15
Yes	6 (14.6)	9 (29.0)	
Residual cancer burden (%)			
0	6 (14.6)	10 (32.3)	0.33
I	4 (9.8)	4 (12.9)	
II	5 (12.2)	4 (12.9)	
III	15 (36.6)	6 (19.4)	
Missing	11 (26.8)	7 (22.6)	
Margin resection (%)			
R0	36 (87.8)	28 (90.3)	1.00
R1	5 (12.2)	3 (9.7)	
Recurrence (%)			
No	33 (80.5)	25 (80.6)	0.97
Yes	8 (19.5)	6 (19.4)	
Hepatic relapse (%)			
No	40 (97.6)	29 (93.5)	0.57
Yes	1 (2.4)	2 (6.5)	
Pulmonary relapse (%)			
No	40 (97.6)	30 (96.8)	
Yes	1 (2.4)	1 (3.2)	1.00
Lymph nodes relapse (%)			
No	37 (90.2)	28 (90.3)	
Yes	4 (9.8)	3 (9.7)	1.00
CNS relapse (%)			
No	40 (97.6)	28 (90.3)	0.21
Yes	1 (2.4)	3 (9.7)	
Local relapse (%)			
No	39 (95.1)	30 (96.8)	1.00
Yes	2 (4.9)	1 (3.2)	
Bone relapse (%)			
No	39 (95.1)	29 (93.5)	1.00
Yes	2 (4.9)	2 (6.5)	
Ovary relapse (%)			
No	41 (100)	30 (96,8)	0.43
Yes	0 (0.0)	1 (3.2)	
Pleural relapse (%)			
No	40 (97.6)	31 (100%)	1.00
Yes	1 (2.4)	0 (0.0)	

Most patients were postmenopausal; 56.1% and 54.8% in the HER2-low and HER2-0 subgroup, respectively. Ductal/non-special type histology was prevalent in both subgroups; 75.6% in the HER2-low and 83.9% in the HER2-0 subgroup and there was no statistical difference regarding differentiation grade between the subgroups.

Significant differences between HER2-low and HER2-0 tumors were detected for HR status and Ki67. Thus, in the HER2-low subgroup, the majority (70%) of patients’ tumors were luminal, and in the HER2-0 subgroup, most patients (64.5%) had TNBC, for a significance level of 0.05 (p = 0.03). Accordingly, with regard to estrogen and progesterone receptors, there were statistically significant differences with p = 0.00 and p = 0.02, respectively, between the two subgroups. The median Ki67 rate was higher in the HER2-0 subset (mean rank = 43.9) compared to HER2-low (mean rank = 30.9) and this difference was statistically significant (p = 0.00).

Clinical T2/T3 were the most prevalent, with homogeneous distribution between the two groups. The proportion of clinical N0 was higher in the HER2-0 subgroup (51.6%) and clinical N1 was higher in HER2-low patients (46.3%), without statistical relevance. However, HER2-low patients had mostly stage III tumors (65.9%) and HER2-0 patients were mainly stage II (61.3%) and this difference was statistically significant (p = 0.03).

Anthracyclines followed by taxanes were the preferred NACT regimen, with doxorubicin plus cyclophosphamide (AC) followed by paclitaxel (P) being the most used regimen; 73.2% in HER2-low and 90.3% in HER2-0 subsets, with the highest proportion of dose-dense regimen being used in HER2-0 patients (54.8%), with no statistical value.

Modified radical mastectomy was the most performed surgery with a greater proportion in the HER2-low subgroup (63.4%) compared to the HER2-0 subgroup, probably reflecting the higher percentage of clinical T3/T4 and clinically positive axillary lymph nodes in the HER2-low subgroup. Resection R0 was more frequent in both subgroups with a uniform distribution of 87.8% vs. 90.3% in the HER2-low and HER2-0 subgroups correspondingly. Concerning residual disease, the RCB III category was the most expressive in the two subgroups, especially in the HER2-low group with 36.6% of cases, although RCB was not determined in 26.8% of the study patients.

**Figure 1:**
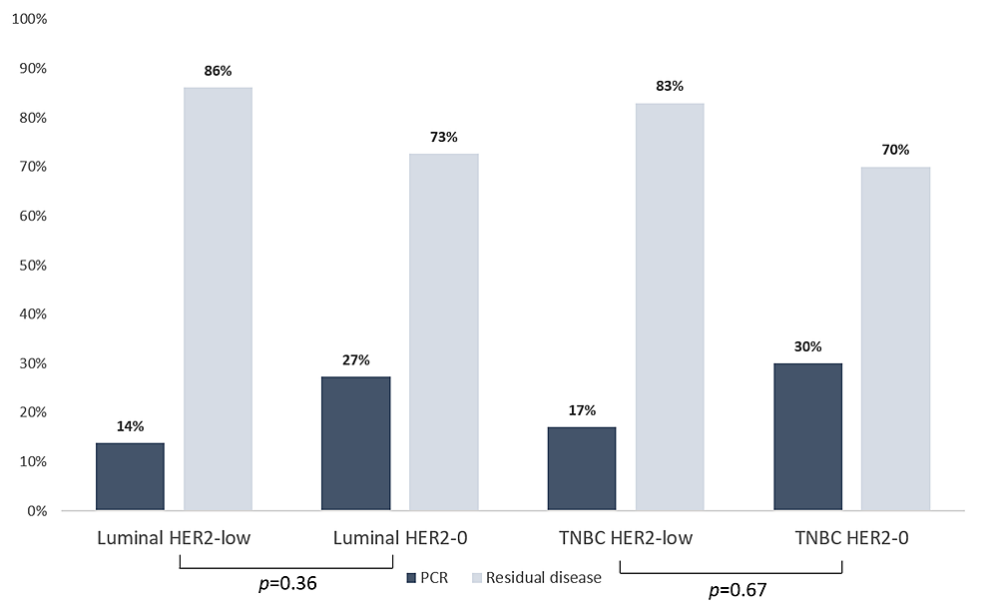
Pathological complete response (pCR) within hormone receptor (HR) groups. Luminal HER2-low (n = 4) vs. luminal HER2-0 (n = 3) and TNBC HER2-low (n = 2) vs. TNBC HER2-0 (n = 6). Fisher’s exact test was used to compare frequencies. HER2 – human epidermal growth factor receptor 2; TNBC – triple-negative breast cancer.

Using ypT0/is ypN0 definition, pCR was obtained in 14.6% of HER2-low vs. 29.0% of HER2-0 patients, a difference not statistically significant (p = 0.15), although clinically relevant (twice the pCR in the HER2-0 group). Within the HER2-0 subgroup, TNBC achieved relatively higher pCR (19.3%) compared with the luminal HER2-0. However, the analysis within HR groups, luminal HER2-low vs. luminal HER2-0 and TNBC HER2-low vs. TNBC HER-0, showed no statistically significant differences, p = 0.36 and p = 0.67, respectively (Figure 1). Additionally, we did not find any relevant differences in pCR between HER2 IHC 1+ and HER2 IHC 2+/non-amplified tumors.

**Figure 2:**
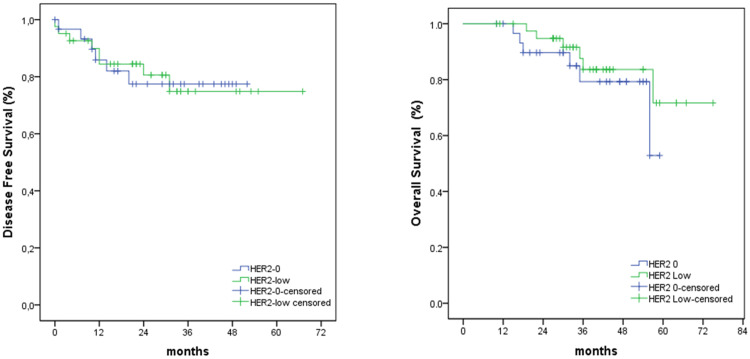
Disease-free survival and overall survival according to HER2-low and HER2-0. Survival curves were estimated using the Kaplan-Meier method and compared with the log-rank test. HER2 – human epidermal growth factor receptor 2.

There were 19.4% recurrences in the general study with equivalent proportions in the two groups (p = 0.97) and there was no relevant difference regarding DFS and OS between the subgroups (p = 0.97 and p = 0.35, respectively). DFS rates assessed at six and 12 months were slightly higher in the HER2-low group, respectively, 97% and 93% compared to 86% and 84% in the HER2-0 group. Assessing at 18 months and 24 months revealed similar results, DFS rate 82% versus 84% respective to HER2-low and HER2-0. Differences were even less evident in OS (Figure 2). There was no death event reported in the 12 months after diagnosis and survival rates at 18 and 24 months were 100% vs. 90% and 95% vs. 90% for HER2-low and HER2-0 subgroups, respectively.

## Discussion

The binary categorization of HER2 status has been recently questioned by emerging evidence on the impact of new ADC targeting HER2 among HER2 BC with advanced HER2-low disease [7,11-13]. A number of studies emerged in the past years have identified differences between HER2-low and HER2-0 disease, currently classified as HER2− disease, suggesting that HER2-low tumors constitute a distinct nosological entity [6-8]. In this study, the HER2-low subgroup was prevalent representing 56.9% of all patients, which is in agreement with previous reports, whose frequency ranged from 45% to 55% [6,8,14,16]. There was a slightly higher proportion of HER2 2+/ISH non-amplified in our study, which is opposite to the previous series where the vast majority of HER2-low patients had an IHC 1+. However, the difference was not relevant statistically considering that our study included a small number of patients to address these parameters accurately.

The results were consistent with previous data, concerning HR expression, HER2-low tumors expressing a higher level of HR (65-83%) [6-8,14]. Specifically, a study about molecular analysis including PAM50 gene expression features of HER2-low BC found that ERBB2 levels are predominant in HR+/HER2-low tumors compared with TNBC/HER2-low tumors [6]. Indeed, in our cohort, a large majority of HER2-low patients (70%) had HR+ tumors and patients' HER2-0 tumors were mainly TNBC and this was statistically relevant. Thus, HR status has an important role in HER2-low disease and these findings may corroborate the assumption that HER2-low and HER2-0 may represent two distinct clinical entities. Moreover, there was a significant difference regarding proliferation index (Ki67) between the two subgroups, HER2-0 tumors expressing higher Ki67 rate, which can be explained by the higher proportion of TNBC in this subgroup, which is associated with higher expression of Ki67 than non-TNBC [17].

According to other publications [6,18], HER2-low status was associated with more locally advanced tumors, with the majority of stage III patients in this subgroup, statistically relevant compared to HER2-0. HER2-low patients presented with more clinically positive axillary lymph nodes and a slightly higher percentage of clinical T3/T4 tumors. However, it is important to consider that the HER2-low subgroup comprised a large proportion of patients with luminal-like tumors for whom NACT has generally been indicated in locally advanced disease; therefore, these results should be interpreted with caution. In contrast, CT dose-dense regimens were used more often in HER2-0 patients, likely as a reflection of more TN tumors in this pool requiring a more aggressive therapeutic regimen. Regarding surgical treatment, modified radical mastectomy was the most performed surgery, especially in HER2-low patients, which can be explained by the greater number of patients in stage III as previously mentioned.

Although we cannot interpret the results considering the considerable amount of missing data, HER2-low patients had more residual disease after NACT and surgery, which can be justified by having more locally advanced disease and treated with a "less aggressive" CT regimen (i.e., less dose-dense regimen). Despite this, there was no statistically significant difference compared to the HER2-0 subgroup. Our cohort had some limitations; therefore, we recommend further studies.

In our study, HER2-low status did not impact the response rate after NACT with only 14.6% of pCR rate in this subgroup and not statistically significant. Similar results were presented in a retrospective study of 449 TNBC patients. The pCR rate was not significantly different between the HER2-low group versus the HER2-0 group (35.7% vs. 41.8%, p = 0.284) [19]. Another series of 331 patients showed no difference in the pCR rates between receptor hormonal positive HER2-0 and HER2-low subgroups (8% vs 13%) and were non-statistically significant. A higher pCR rate was achieved among TNBC HER2-0 versus HER2-low tumors (56% vs. 39%) although they were equally non-statistically relevant [20].

On the other hand, a pooled analysis from four prospective, neoadjuvant clinical trials of 2,310 patients revealed a lower pCR rate in the HR+ HER2-low group compared to HR+ HER2-0 (17.5% vs. 23.6%, respectively, p = 0.024), and no difference was observed in HR− subgroups [7]. These findings were explained by the association of reduced aggressiveness indicators in the HR+ HER2-low subset such as a lower number of grade III tumors, lower Ki67 status, and a reduced number of TP53 mutations compared with HER2-0 tumors and consequently reduced pCR rate to NACT [7]. Ki67 is a well-known positive predictor of response and the lower level in the HER2-low subgroup in our study is in accordance with the lower pCR in this group [17]. However, this is not true for other factors such as tumor grade; moreover, there was no difference between luminal HER2-low and TNBC HER2-low (p = 0.36) and thus no impact of hormonal receptor in the HER2-low group. Furthermore, despite the chemosensitivity of HER2+ disease, whether HER2-low expression is predictive of response to CT is unclear and further research is needed [10].

Additionally, there was no HER2-low status impact on survival outcomes (Figure 1). The effect of HER2-low status on the prognosis remains debatable among several studies. A number of series corroborate our findings [6,8,14,21] but others suggested worse outcomes for HER2-low in localized node-positive and luminal early BC, although these studies compared HER2-0/HER2 1+ versus HER2 2+/ISH negative patients [18,22]. Another cohort of 3,689 patients with a vast majority of advanced BC did not achieve a prognostic impact for HER2-low status on OS [6].

As previously mentioned, new ADCs are being widely studied in clinical trials in advanced HER2-low BC and to a lesser extent in the early-stage setting [8]. For instance, results of a phase III trial in advanced/metastatic HER2-low BC (NCT03734029/DESTINY-Breast04) are awaited and NCT04553770 clinical trial in HR+ HER2-low early BC is currently recruiting patients to neoadjuvant trastuzumab-deruxtecan (± anastrozole) with pCR for the primary endpoint.

This study has several limitations that need to be addressed, including single institution and retrospective design, a small number of subjects, and a short period of follow-up. Also, different guidelines (ASCO/CAP) for HER2 testing and interpretation may have been used over time, the same for hormonal receptor evaluation, and with the absence of HR and HER2 IHC status, central pathological confirmation and variability between pathologists may have affected the results. Additionally, considering the median follow-up of 35.5 months, the data were immature for estimating the survival rates, and our study represents a very small sample, so the results may be insufficient to draw definitive conclusions.

## Conclusions

The results of this study support that HER2-low may be a distinct biological entity based on the prevalence of hormonal receptors in the early-stage BC setting in accordance with previously published data. No statistical difference in pCR was noted, but the difference was clinically relevant (HER2-low had half the pCR rate versus HER2-0) and this may reflect a limitation of the retrospective nature and small population of the study. Given the new HER2-low targeting ADCs preliminary activity data, HER2-low patients may present a unique subset population for exploring new treatment options for improving BC outcomes and warrant further research.

## Figures and Tables

**Figure 3:**
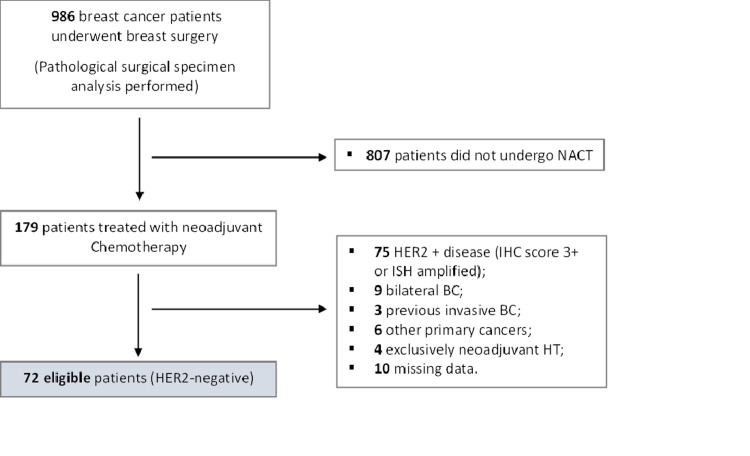
Flowchart for patient selection. NACT – neoadjuvant chemotherapy; HER2 – human epidermal growth factor receptor 2; IHC – immunohistochemistry; ISH – in situ hybridization; BC – breast cancer; HT – hormonal therapy.

## Appendices

Supplementary figure

## References

[1] Tarantino P, Hamilton E, Tolaney SM (2020). HER2-low breast cancer: pathological and clinical landscape. J Clin Oncol.

[2] Schettini F, Prat A (2021). Dissecting the biological heterogeneity of HER2-positive breast cancer. Breast.

[3] Ferraro E, Drago JZ, Modi S (2021). Implementing antibody-drug conjugates (ADCs) in HER2-positive breast cancer: state of the art and future directions. Breast Cancer Res.

[4] Wolff AC, Hammond ME, Allison KH (2018). Human epidermal growth factor receptor 2 testing in breast cancer: American Society of Clinical Oncology/College of American Pathologists clinical practice guideline focused update. J Clin Oncol.

[5] Schettini F, Pascual T, Conte B (2020). HER2-enriched subtype and pathological complete response in HER2-positive breast cancer: a systematic review and meta-analysis. Cancer Treat Rev.

[6] Schettini F, Chic N, Brasó-Maristany F (2021). Clinical, pathological, and PAM50 gene expression features of HER2-low breast cancer. NPJ Breast Cancer.

[7] Denkert C, Seither F, Schneeweiss A (2021). Clinical and molecular characteristics of HER2-low-positive breast cancer: pooled analysis of individual patient data from four prospective, neoadjuvant clinical trials. Lancet Oncol.

[8] Agostinetto E, Rediti M, Fimereli D (2021). HER2-low breast cancer: molecular characteristics and prognosis. Cancers (Basel).

[9] Fehrenbacher L, Cecchini RS, Geyer CE Jr (2020). NSABP B-47/NRG oncology phase III randomized trial comparing adjuvant chemotherapy with or without trastuzumab in high-risk invasive breast cancer negative for HER2 by FISH and with IHC 1+ or 2. J Clin Oncol.

[10] Mutai R, Barkan T, Moore A (2021). Prognostic impact of HER2-low expression in hormone receptor positive early breast cancer. Breast.

[11] Modi S, Park H, Murthy RK (2020). Antitumor activity and safety of trastuzumab deruxtecan in patients with HER2-low-expressing advanced breast cancer: results from a phase Ib study. J Clin Oncol.

[12] Nakada T, Sugihara K, Jikoh T, Abe Y, Agatsuma T (2019). The latest research and development into the antibody-drug conjugate, [fam-] trastuzumab deruxtecan (DS-8201a), for HER2 cancer therapy. Chem Pharm Bull (Tokyo).

[13] Banerji U, van Herpen CML, Saura C (2019). Trastuzumab duocarmazine in locally advanced and metastatic solid tumours and HER2-expressing breast cancer: a phase 1 dose-escalation and dose-expansion study. Lancet Oncol.

[14] de Moura Leite L, Cesca MG, Tavares MC (2021). HER2-low status and response to neoadjuvant chemotherapy in HER2 negative early breast cancer. Breast Cancer Res Treat.

[15] Cortazar P, Zhang L, Untch M (2014). Pathological complete response and long-term clinical benefit in breast cancer: the CTNeoBC pooled analysis. Lancet.

[16] Eiger D, Agostinetto E, Saúde-Conde R, de Azambuja E (2021). The exciting new field of HER2-low breast cancer treatment. Cancers (Basel).

[17] Keam B, Im SA, Lee KH (2011). Ki-67 can be used for further classification of triple negative breast cancer into two subtypes with different response and prognosis. Breast Cancer Res.

[18] Eggemann H, Ignatov T, Burger E (2015). Moderate HER2 expression as a prognostic factor in hormone receptor positive breast cancer. Endocr Relat Cancer.

[19] Domergue C, Martin E, Lemarié C (2021). 156P impact of HER2 status (HER2-low versus HER2-0) on complete histologic response after neoadjuvant chemotherapy in early triple-negative breast cancer (TNBC). Ann Oncol.

[20] Reinert T, Sartori GP, Souza AA (2021). Abstract PS4-22: prevalence of HER2-low and HER2-zero subgroups and correlation with response to neoadjuvant chemotherapy (NACT) in patients with HER2-negative breast cancer. Cancer Res.

[21] Horisawa N, Adachi Y, Takatsuka D (2021). The frequency of low HER2 expression in breast cancer and a comparison of prognosis between patients with HER2-low and HER2-negative breast cancer by HR status. [PREPRINT]. Breast Cancer.

[22] Rossi V, Sarotto I, Maggiorotto F (2012). Moderate immunohistochemical expression of HER-2 (2+) without HER-2 gene amplification is a negative prognostic factor in early breast cancer. Oncologist.

